# Supplementary material for: Multimodal imaging and functional analysis of the chick NMDA retinal damage model
Source: PLoS One. 2021 Sep 7;16(9):e0257148. doi: 10.1371/journal.pone.0257148 (PMC8423281; doi:10.1371/journal.pone.0257148)
Supplement: S1 File — (ZIP) [file pone.0257148.s004.zip › SD-OCT/C200727/007/D21-Pwk4/C200727-007_OS_R_14_0_REGAVG0000107/Manual_PreDefineThreeByFourGridRadialRetina_1.pdf]

Three By Four Grid Radial Retina Template Report

**Patient ID**	C200727	**Name**	See InVivoVue
**Exam Date**	See InVivoVue	**Exam Time**	See InVivoVue
**Eye**	OS	**Averaged**	N/A
**Depth (mm)**	1.65	**Depth Samples**	1024
**Pitch (um/pix)**	14.000 x 2333.333 x 1.608	**Refractive Index**	See InVivoVue
**Scan Type**	Radial 14.000mm x 14.000mm x 1.647mm , 1000 x 6 x 1024		

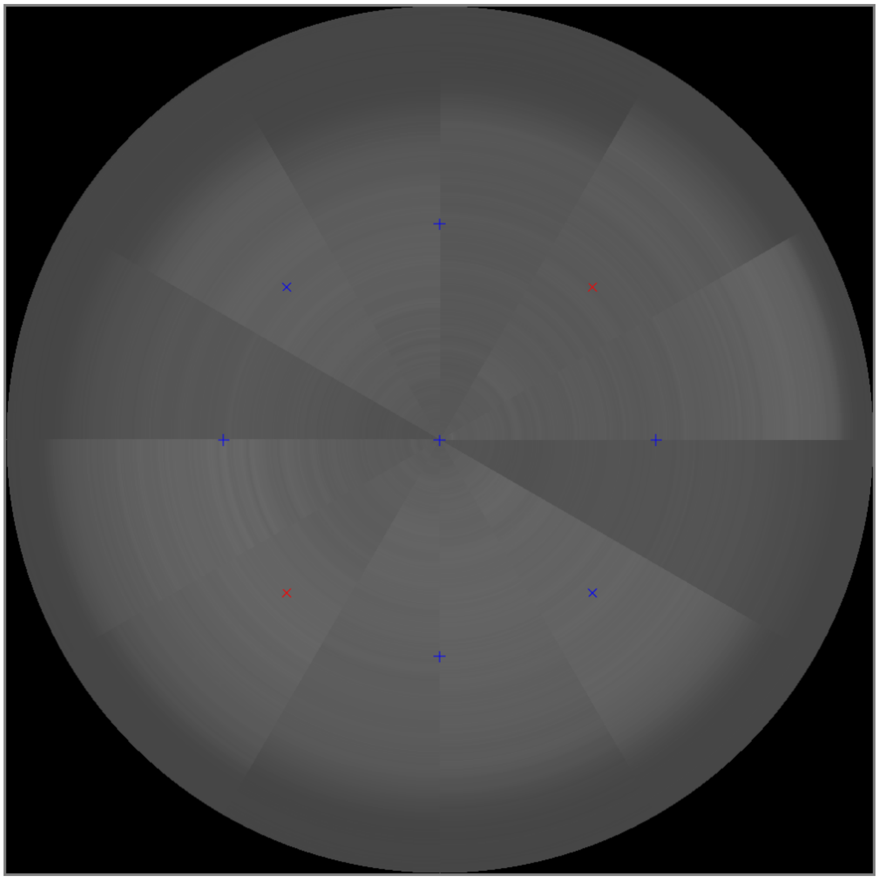

Thickness Metrics

Analysis	Average(mm)	Minimum(mm)	Maximum(mm)	STDEV(mm)
**IRNFL - RPE**	0.2288	0.2197	0.2365	0.0062
**IRNFL - ORNFL**	0.0347	0.0218	0.0587	0.0133
**ORNFL - OPL**	0.1123	0.0899	0.1254	0.0141
**OPL - ETPRS**	0.0622	0.0574	0.0674	0.0035
